# Intubating Ebola Patients: Technical Limitations of Extensive Personal Protective Equipment

**DOI:** 10.5811/westjem.2015.10.28779

**Published:** 2015-12-14

**Authors:** Warren Wiechmann, Shannon Toohey, Cassandra Majestic, Megan Boysen-Osborn

**Affiliations:** University of California, Irvine, Department of Emergency Medicine, Irvine, California

As hospitals across the nation were preparing for the possibility of Ebola or Middle Eastern respiratory syndrome (MERS-CoV) cases, healthcare workers underwent intricate training in the use of personal protective equipment (PPE). An Ebola or MERS-CoV patient requiring intubation places a healthcare worker at risk for exposure to bodily secretions. The procedure must be performed only after appropriate PPE is donned.^1^ Intubating while wearing PPE is yet another challenge identified in caring for these patients. Manual dexterity and free movement decreases when wearing PPE, and may increase length of time to successful intubation.

We elicited the opinion of subjects performing direct laryngoscopy versus video-assisted laryngoscopy on manikins while wearing PPE. Additionally, we recorded multiple intubation attempts by these clinicians using Google Glass. Two PPE-donned clinicians both agreed that intubation was not technically different between direct versus video-assisted techniques. However, the subjects felt that direct laryngoscopy was noticeably more labor intensive than the video-assisted technique. Subjects also felt more temperature-related discomfort during direct laryngoscopy. For one subject, contamination was more common during direct laryngoscopy, when the PPE hood contacted the patient’s face or endotracheal tube. From this simulation experience, we recommend video laryngoscopy as a preferred method of intubating a patient while donning PPE.

## Figures and Tables

**Video:** Ebola intubation video.
